# Novel small fragment removal system may improve extraction of renal calculi: an in vitro study

**DOI:** 10.1007/s00345-024-05377-4

**Published:** 2024-12-06

**Authors:** John Lazarus, Mark Wellman, Jørgen Wulfsberg, Tommaso Ceccato, Jeff John

**Affiliations:** 1 Division of Urology, Groote Schuur Hospital, University of Cape Town, Cape Town, South Africa; 2 Faculty of Medicine and Health Sciences, Norwegian University of Science and Technology, Trondheim, Norway; 3 Department of Surgery, Oncology, Gastroenterology and Urological Unit, University of Padova, Padova, Italy; 4 Department of Urology, Frere Hospital, Walter Sisulu University, Mthatha, South Africa

**Keywords:** Renal calculi, Ureterorenoscopy, Intrarenal pressure, Ureteral access sheath, Endourology

## Abstract

**Objective:**

To describe a novel Small Fragment Removal System (SFRS) which is hypothesized to improve stone fragment removal during flexible ureteroscopy in patients with urolithiasis. The SFRS consists of three parts: a Syphon Ureteric Access Sheath (SUAS), a Dual Action Pump (DAP) and an Agitator. This bench assessment aims to assess the SFRS’s impact on intra-renal pressure (IRP), irrigant flow rate and stone fragment removal compared to a traditional UAS.

**Materials and methods:**

A validated phantom kidney and fibre optic pressure sensor was used to assess IRP. Standardized irrigation via a flexible ureterorenoscope was instilled through a traditional UAS and compared to the novel SFRS. Both were 11/13Fr in size. Measured minute volume, IRP and percentage of stone fragments removed were compared.

**Results:**

The mean IRP using a traditional UAS and SFRS was 24,3mmHg and 9,4mmHg respectively. The flow rate of the traditional UAS was 25mL/min, compared to 31mL/min with the SFRS attached. During bolus fluid administration using the traditional UAS the maximum IRP increased to 41mmHg, compared to 9,3mmHg with the SFRS attached. A mean of 42,7% (0,103 g of 0,305 g) of weighed dry stones were removed with the traditional UAS compared to 77,6% (0,233 g of 0,299 g) with the SFRS attached, *p* = 0,017.

**Conclusions:**

The described Small Fragment Removal System (SFRS) is different from traditional UASs by incorporating a syphoning mechanism. In addition, it has a Dual Action Pump which both boluses and augments the aspiration of irrigant by the Syphon. It further includes a deflectable Agitator to flush out stone fragments.

**Supplementary Information:**

The online version contains supplementary material available at 10.1007/s00345-024-05377-4.

## Introduction

The urologist’s primary goal is to render a patient with urolithiasis stone-free. Despite advances in endourology techniques and technology, residual stone fragments persist potentially necessitating further surgery and increasing the likelihood for subsequent clinical stone episodes.

A recent Cochrane metanalysis of the natural history of fragments following stone surgery revealed that the intervention rate for fragments measuring ≤ 4 mm was 22% at 50 months, whereas for fragments measuring > 4 mm, the intervention rate was 47%. The disease progression rate for fragments measuring ≤ 4mm was 47% while for fragments measuring > 4mm, it was 88% [1]. These disappointing Cochrane review findings have led some commentators to conclude that the term “clinically insignificant” when used to describe residual stone fragments after stone surgery, is likely a misnomer [2].

The objective of this paper is to describe the design of a novel Small Fragment Removal System (SFRS). The SFRS is designed to wash out small fragments during and immediately after stone fragmentation by using pulsatile and simultaneous irrigation and suctioning. This is achieved by a manually operated dual action pump (DAP) that may be handled by either foot or hand. The suctioning function of the DAP pump is augmented by a previously described Syphon Ureteric Access Sheath (SUAS). This sheath demonstrated the potential to improve the irrigant flow while reducing the intrarenal pressure (IRP) during flexible ureteroscopy (fURS) [3, 19].

We further document two laboratory experiments to investigate the in vitro performance of the SFRS. The objective of these experiments was to assess whether the SFRS can remove small stone fragments at a low IRP – a proxy for the safety of the SFRS. Additionally, the efficacy of the SFRS was assessed by determining the proportion of small fragments that were successfully removed. It is hypothesised that the system would remove more than 75% of stone fragments.

## Materials and methods

### Design of the small fragment removal system (SFRS)

**Fig. 1:**
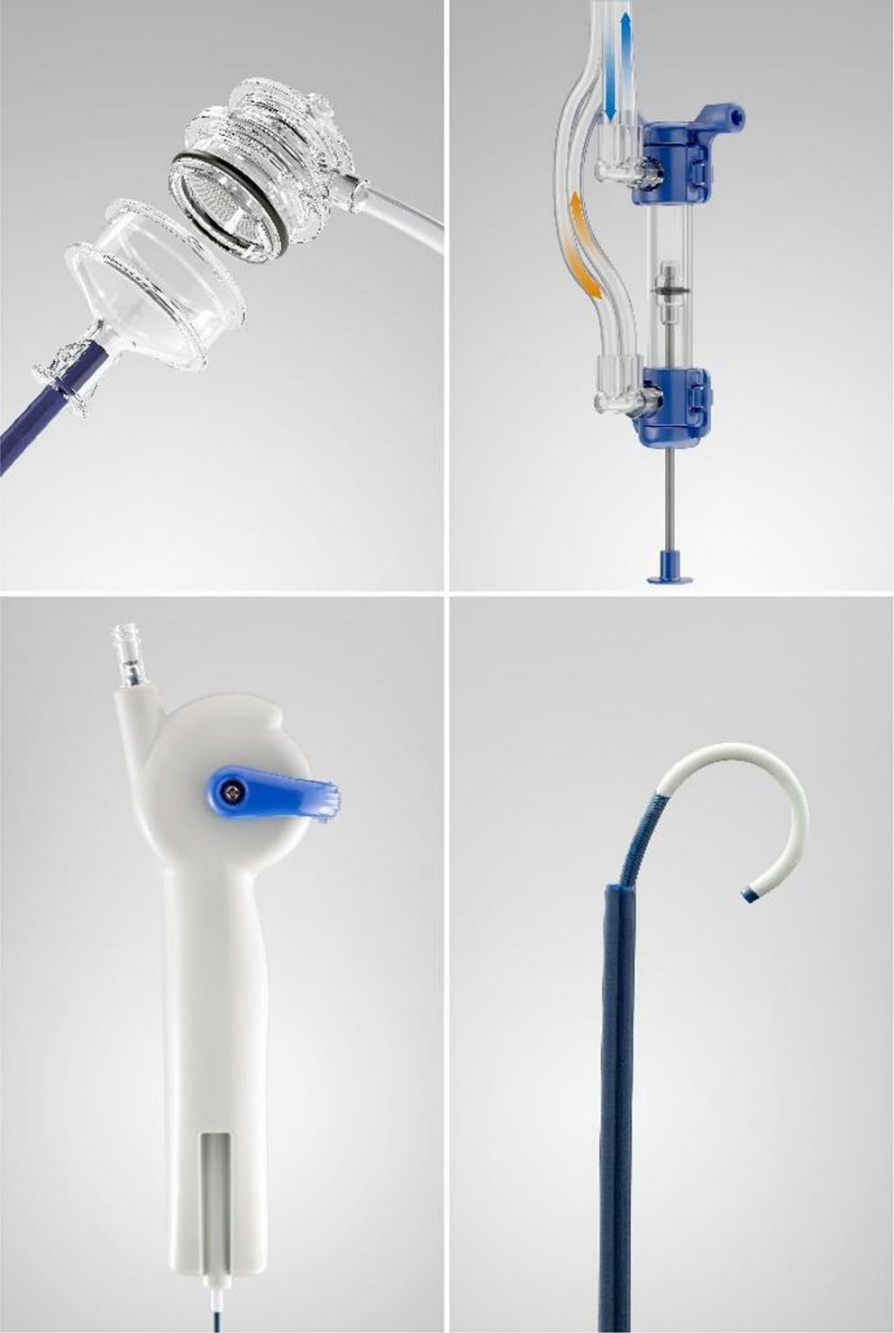
The syphon ureteric access sheath (upper left), Dual action pump (upper right) and 6 French agitator (bottom left and bottom right)

The SFRS comprises three separate components: the Syphon Ureteric Access Sheath (SAUS), a Dual Action Pump (DAP), and an Agitator incorporated into one system (see Fig. 1).

#### Syphon ureteric access sheath (SAUS)

This novel ureteric access sheath (UAS), previously described by Lazarus [3] and Yekani [19] incorporates a syphon mechanism at the outlet of an otherwise traditional UAS, improves irrigant outflow, and reduces IRP compared to a traditional UAS [19].

#### Dual action pump (DAP)

This is a low-volume, user-controlled (foot or hand) pumping unit that enables the urologist to deliver fluid boluses of no more than 2mL, into the upper urinary tract as the unit is compressed, while the same volume of fluid is drawn out. The greater the compression, the larger the bolus volume; the quicker the compression, the faster the bolus is jetted into the upper urinary tract. The delivery of a bolus during DAP compression momentarily renders the small stone fragments waterborne. These fragments are expelled from the collecting system simultaneously by aspiration of the same volume of bolus from the kidney through the descending arm of the SAUS. Bolus delivery and bolus aspiration are synchronised in time to prevent changes in the IRP. The DAP is released to prime the pump to deliver the next bolus. Repetitive, careful compression and release can be performed in quick succession to quickly clear away debris. Flushed-out fragments are collected in a sieve inside the syphon box, allowing easy retrieval.

#### Agitator

This thin (6 Fr) steerable catheter enables urologists to direct fluid boluses in the direction of stone fragments in the kidney. The Agitator is steered in the same way as a standard flexible ureterorenoscope by moving the thumb lever up and down and rotating the Agitator. A radio-opaque marker band at the tip of the Agitator enables the surgeon to navigate under fluoroscopic guidance. At the entrance to each calyx, a few boluses are delivered to make any stone fragments in the calyx waterborne to allow them to be aspirated out, with the action of the DAP.

### In-vitro assessment

#### Safety assessment

**Fig. 2:**
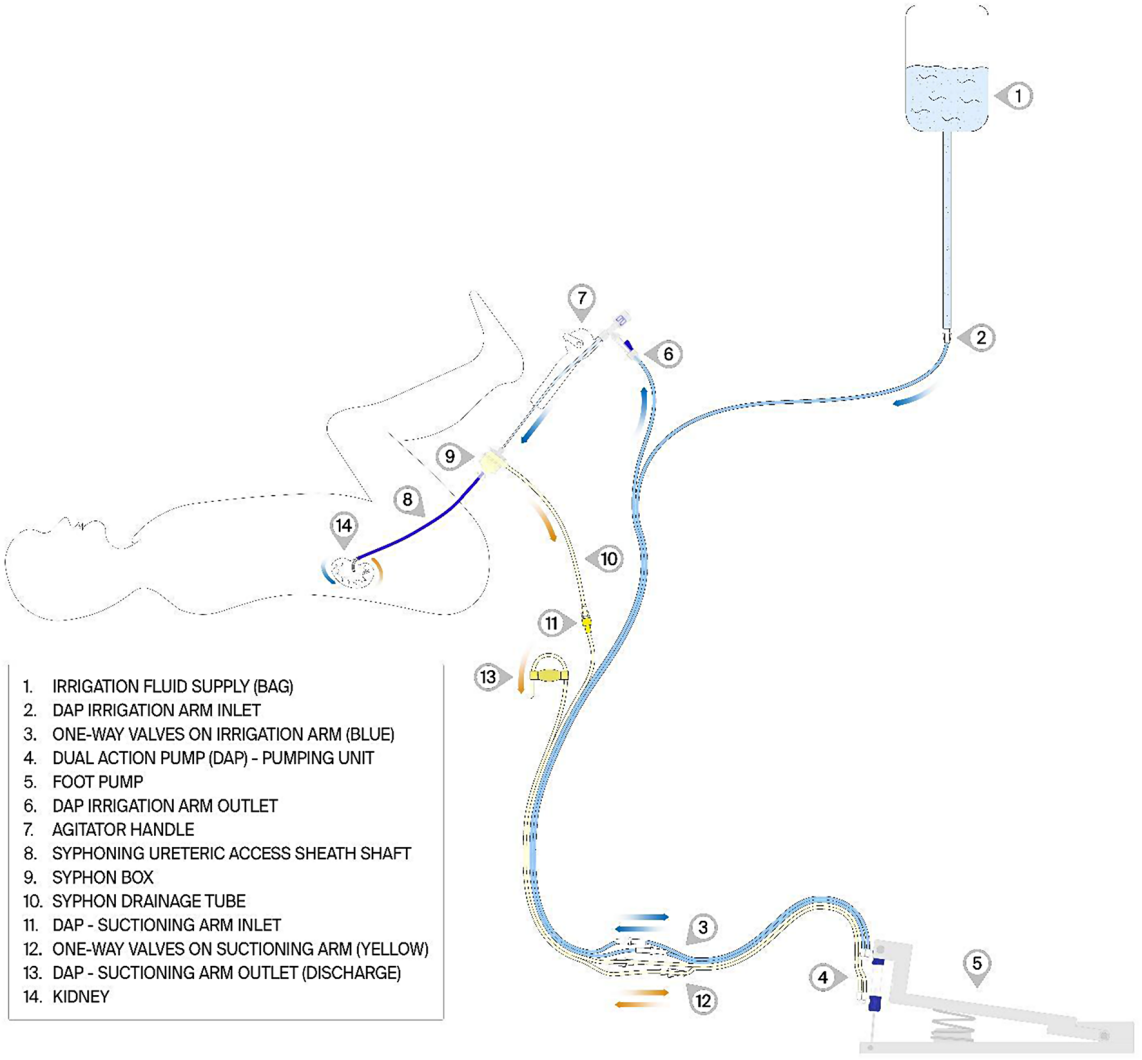
The small fragment removal system (SFRS) schematic. It shows the sub-devices and how irrigant flows from a suspended bag through a flexible ureterorenoscope to the upper urinary tract (phantom kidney). The fluid is then removed via the Syphon UAS. Irrigation and removal of fluid is augmented if the Dual Action Pump (DAP) is activated by the foot pump

To evaluate safety, an experimental model was established according to the layout shown in Fig. 2. Notably, a fully validated phantom kidney, three-dimensionally printed from a high-resolution MRI scan (Max Planck Institute for Intelligent Systems, Stuttgart, Germany), was used to assess the safety of the device [4]. This kidney has demonstrated the ability to accurately mimic the anatomy and tensile/compliance properties of the human kidney, and would therefore provide an accurate reflection of the IRP that one expected in vivo. These IRP measurements were obtained using a fibre optic pressure sensor calibrated to atmospheric pressure and inserted into the renal pelvis through a micro-nephrostomy. To avoid leakage from the micro-nephrostomy, a Tuohy-Borst adapter was employed, and the entry point of the micro-nephrostomy was further sealed with silicon. A FISO FOP-M200 model with a diameter of 200 μm, equivalent to 0.008 inch (FISO Technologies Inc., Quebec City, Canada) was used. The FISO pressure metre has a sampling rate of 250 Hz.

A conventional cysto-irrigation set was connected to the DAP irrigation arm inlet positioned at a column height of 110 cm (81 mmHg), while the DAP suctioning arm outlet was suspended 40 cm below the phantom kidney model (see Fig. 2). The air was removed from the phantom kidney, an f-URS (Flex X2, Karl Storz, Tuttlingen, Germany) was inserted into the kidney, and gravitational irrigation commenced.

For the experiment, with the SFRS connected, the IRP was measured constantly for 30 s, from which the mean IRP was calculated. Second, the irrigant flow volume was measured for 1 min. Lastly, five DAP boluses were administered 1 s apart, and the maximum IRP was recorded. To establish a control, the above procedure was repeated without the use of SFRS employing a conventional UAS, where the DAP was only capable of delivering the irrigant.

#### Efficacy assessment

A separate translucent kidney model (Max Planck Institute for Intelligent Systems, Stuttgart, Germany), made from silicone and moulded based on a high-resolution MRI image, was used to determine the efficacy of the SFRS. The selection of this model was based on its translucency, which enabled clear visualisation of the stone fragment removal process.

Small sandstone fragments, which were utilised to stimulate kidney stone fragments, were filtered through a sieve to ensure that the size of all fragments ranged from 0.8 to 1.3 mm. Dried, weighted (0.30 g) stone fragments were measured using a calibrated Mettler analytical scale and inserted into the collecting system of the clear kidney model using a syringe with fluid and an open-ended catheter. During the experiment, the SFRS was connected, and the Agitator was inserted through the SUAS into the kidney model. Irrigation flow was then started. The DAP was activated, and the Agitator was steered under fluoroscopic guidance to extract as many stone fragments as possible from the collecting system. Short breaks were periodically taken to inspect the collecting system. Following a period of 180 s of DAP, all actions were halted. The small expelled stone fragments were collected, dried, and weighed. The weight of the recovered fragments was compared to the initial 0.30 g introduced before intervention and expressed as a percentage. For the control, the same procedure was repeated with a conventional UAS and manual pump directing fluid boluses to the stone fragments. Similarly, the weight of the collected fragments in the control was also compared to the initial 0.30 g introduced before intervention and expressed as a percentage. The investigator was blinded during the procedure and was unaware of whether the SFRS was connected.

The study was approved by the Surgical Departmental Research Committee of the University of Cape Town (reference: 2023/127). Statistical analysis was performed using the GraphPad Prism version 5.03 software. Paired t tests were used. Statistical significance was set at *p* < 0.05.

## Results

### Safety

**Fig. 3:**
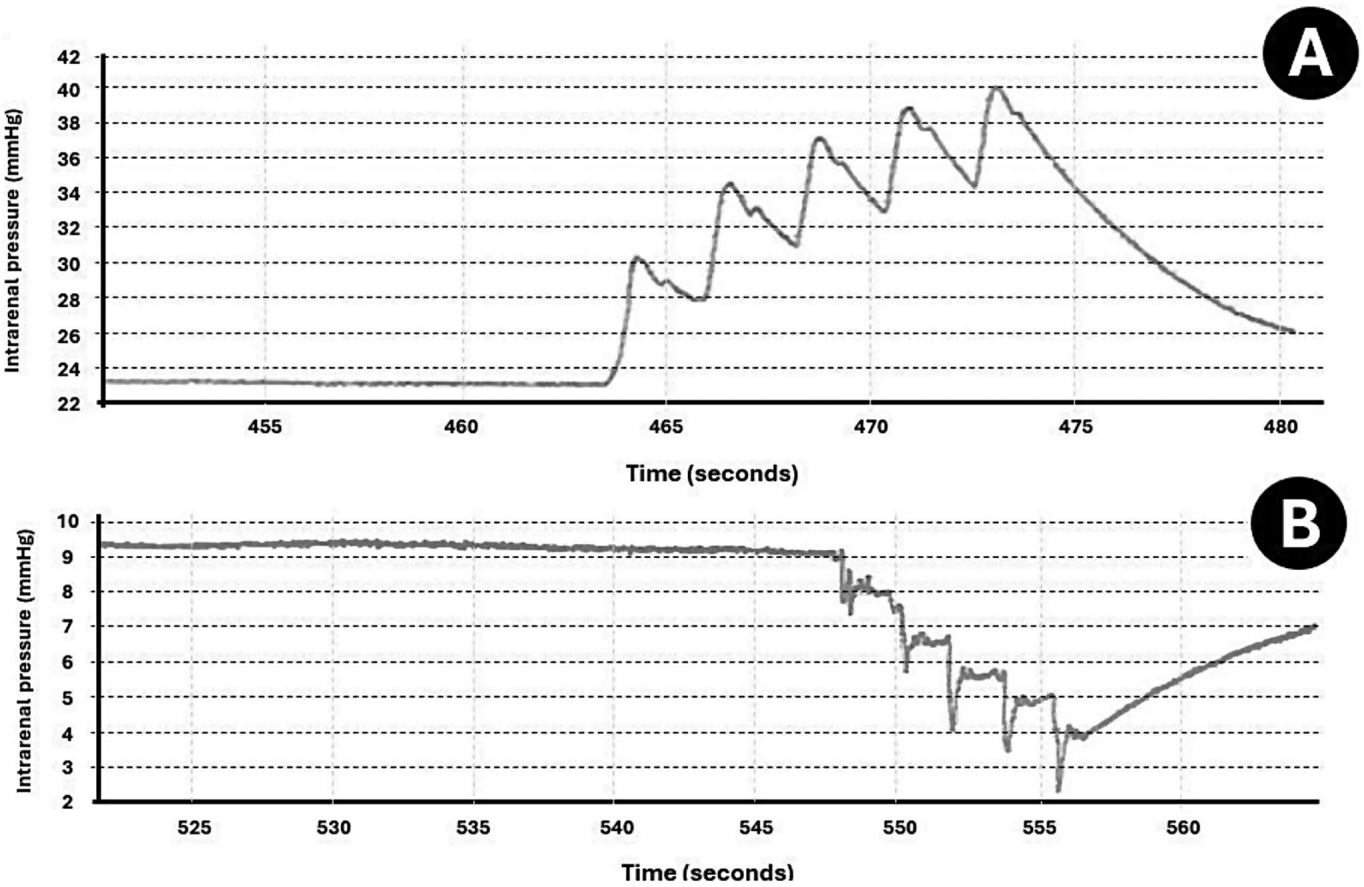
IRP measurements taken during the fluid bolus assessment. (**A**) shows that for a traditional UAS there is a rise in IRP, while in (**B**) the SFRS shows a decline in IRP during periods of vigorous and repeated DAP actuation

The baseline IRP with a conventional UAS was 24,3 mmHg, and 9,4 mmHg when the SFRS was attached. The flow rate (minute volume) of conventional UAS was 25 mL/min. The flow rate was increased to 31 mL/min with the attached SFRS. While administering fluid boluses with the foot pump (five boluses each given 1 s apart), the maximum IRP increased to 41 mmHg when using the conventional UAS. In contrast, the maximum IRP was 9,3 mmHg when boluses were administered with the attached SFRS. No instances of IRP above the “conventionally safe” 40 mmHg pressure limit were observed for the SFRS. Figure 3 displays the IRP readings recorded while administering fluid boluses. For the conventional UAS, the IRP consistently increased from one bolus to the next (Fig. 3A). However, in the SFRS, the IRP decreased despite periods of vigorous and repeated DAP activation (Fig. 3B).

### Efficacy

**Fig. 4:**
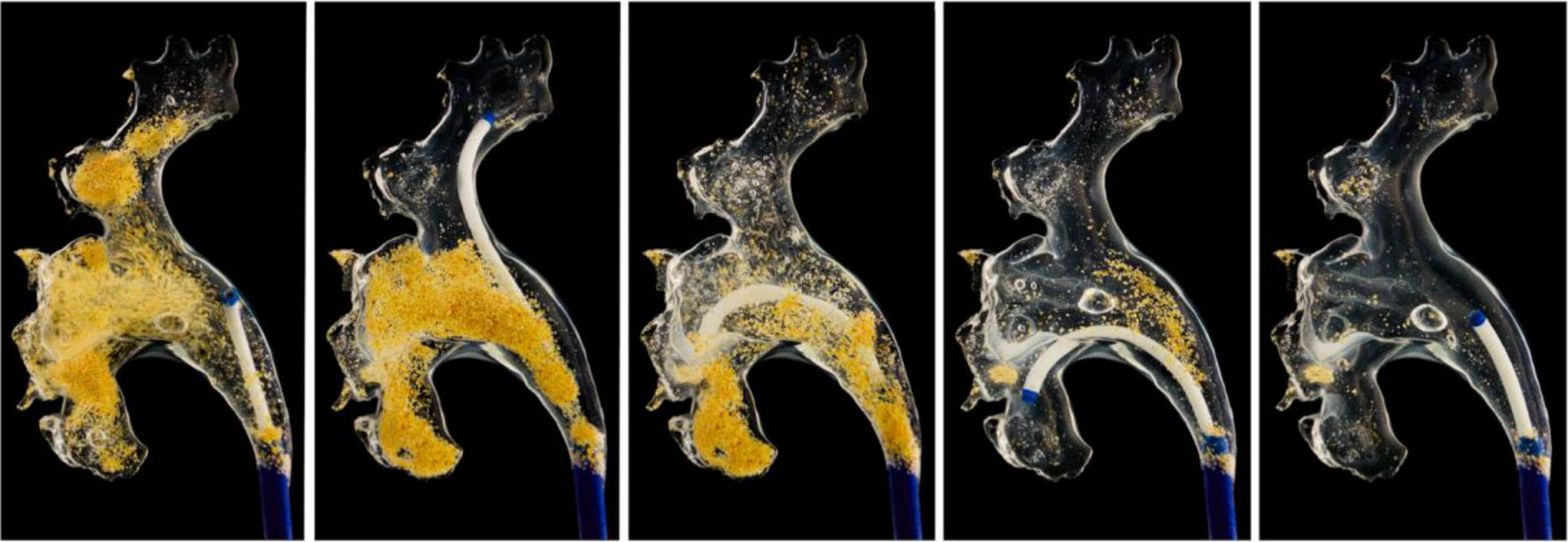
Photograph of the clear kidney model before and after small fragment removal using the SFRS

The SFRS was able to remove a mean of 77,6% (0,233 g of 0,299 g) of the fragments. In comparison, 42,7% (0,103 g of 0,305 g) of the fragments were extracted without SFRS (*p* = 0,017) See Fig. 4.

## Discussion

Despite advances in endourology techniques and technology obtaining a “stone free” status for patients with urolithiasis remains a challenge.

Emerging literature illustrates the fate of these “clinically insignificant fragments”. A recent Cochrane metanalysis of the natural history of fragments following stone surgery showed an intervention rate for ≤ 4 mm fragments of 22%, while for > 4 mm fragments it was 47% at 50 months. Disease progression rates for ≤ 4 mm was 47% and > 4 mm 88% at 50 months [1].

Streem et al. have argued that the notion of “clinically insignificant fragments” is a misnomer and should be abandoned [2].

Given the disappointing results of the Cochrane metanalysis the authors concluded that patients with residual fragments should be appropriately counselled to allow informed decision-making regarding potential further management [1].

An editorial commenting on the Cochrane metanalysis reminds that “the goal of treatment for kidney stones should therefore be to obtain a stone-free status and minimize repeat interventions, particularly considering we do not fully understand the impact of repeat interventions on patients’ quality of life” [6].

In addition to quality of life, the economic impact of stone disease recurrence needs to be considered. Geraghty et al. have shown that in the United Kingdom the additional cost nationally (of treating stone fragments or recurrence) following an initial stone episode is estimated to rise by up to 85% by 10 years. This translates to an estimated GBP 324 million annual cost [7].

Modern ureteroscopy and laser technology can render stones into very small fragments via “dusting” techniques - laser vaporization modes using high frequency (15–20 Hz) and low energy (0.5–1.0 J). This raises the question if these < 1 mm fragments can be labelled “insignificant”.

The fate of these dusts has been studied by Kang et al. [5]. Their study showed that remnant particles persisted in 60% of patients with dusts. They concluded that any size of post-treatment fragment has the potential to become “clinically significant”. They recommended taking time to basket or wash out dust particles.

This paper described a novel Small Fragment Removal System. The in vitro assessment documented here suggests that the device holds clinical potential. The Agitator device removed 77,6% of small fragments, 35% more than when a traditional UAS was used. It achieves this by the combined aspiration of irrigant created by the Syphon UAS and separately by the foot activated Dual Action Pump which both boluses and aspirates.

We have also demonstrated safe intra renal pressures with the SFRS and increased irrigant flow. This increased irrigant flow may hold clinical potential to improve visibility for the urological surgeon.

The urological literature emphasises the importance of maintaining safe intrarenal pressures during fURS. Elevated IRP during fURS is a known risk factor for not only urosepsis, but also pain, haemorrhage and acute kidney injury [11]. Zhong et al. studied infective complications and demonstrated in a series of 260 patients undergoing fURS that a systemic inflammatory response was seen in 8.1% [15].

The normal IRP is in the range of 0–15 mmHg (0–20 cmH_2_O) [8]. Pyelo-venous reflux has historically, from the original work by Hinman and Lee-Brown [9] been quoted to occur above 40 mmHg (54 cmH_2_O). More recent studies suggest it can occur at an even lower level of 20 mmHg (27 cmH_2_O) [10].

fURS is known to increase IRP to 60–100 mmHg in the absence of a UAS. If irrigation is forced, pressure rises of > 300 mmHg are not unusual [11]. In a recent study, Jung and Osther [11]. demonstrated in vivo that during routine fURS in 12 patients, the IRP averaged 54 mmHg and pelvic pressure peaks of up to 328 mm Hg occurred. In a 5-min standardised period of simple fURS, 83 pressure peaks of > 50 mmHg were measured.

A variety of methods and devices are described to improve elimination of retained stone fragments. Examples include the following:

1. Various novel stone retrieval basket and grasper designs are described for more efficient stone extraction [12].
2. Devices to prevent ureteral stone retropulsion during intracorporeal lithotripsy such as the “stone cone” [13]. or the “backstop” thermosensitive polymer [14].
3. Other investigators have proposed systems to achieve improved aspiration. Du et al. described a perfusion and suctioning platform and modified UAS. That system monitors IRP under negative-pressure suctioning, thus improving flow. They were able to demonstrate improved patient safety by documenting reduced septic marker elevation in those treated with the system [16].
4. Zhu et al. designed a UAS with an additional suction port, which was attached to a vacuum device. They demonstrated improved stone-free rates, reduced infectious complications, and shorter operative time [17].
5. Lastly, a comprehensive review of techniques to minimise fragments by Hein et al. includes other techniques such as advances in laser technology and robotics [18].

The present study has certain limitations:

1. The work was done in vitro with a phantom kidney, which limits its applicability to live human subjects even though it mimics the compliance of a human kidney. The lack of ureteric peristalsis and the pressure from surrounding tissues differs from a clinical setting. By contrast, the “clear kidney phantom” used to assess stone fragment removal is not physiologically compliant which may impact the applicability of these results. But no results were derived from this clear kidney.
2. Accurate measurement of stone fragments removed could be impacted by stone losses in dealing with the fine sub 1 mm size fragments.
3. Lastly, the small number of experimental attempts to assess safety and efficacy could limit its reproducibility in vivo.

## Conclusions

The described Small Fragment Removal System is different from traditional UASs by incorporating a syphoning mechanism. In addition, it has a Dual Action Pump which both boluses and augments the aspiration of irrigant by the Syphon. It further includes a deflectable Agitator to flush out stone fragments.

Our present in vitro assessment demonstrates that the novel SFRS holds clinical potential to improve patient safety by reducing IRP with potential improved visibility via increased irrigant flow. Lastly, the SFRS was able to remove more than 75% of stone fragments.

These promising findings support further investigation of the SFRS in a pilot clinical trial.

## Electronic supplementary material

Below is the link to the electronic supplementary material.

**Supplementary Material 1:** Demonstration of the Agitator’s elimination of small stone fragments (right) compared to a tractional UAS (left)

## Data Availability

No datasets were generated or analysed during the current study.
